# Does Shape Discrimination by the Mouth Activate the Parietal and Occipital Lobes? – Near-Infrared Spectroscopy Study

**DOI:** 10.1371/journal.pone.0108685

**Published:** 2014-10-09

**Authors:** Tomonori Kagawa, Noriyuki Narita, Sunao Iwaki, Shingo Kawasaki, Kazunobu Kamiya, Shunsuke Minakuchi

**Affiliations:** 1 Gerodontology and Oral Rehabilitation Department of Gerontology and Gerodontology, Graduate School of Medical and Dental Sciences, Tokyo Medical and Dental University, Tokyo, Japan; 2 Department of Removable Prosthodontics, Nihon University School of Dentistry at Matsudo, Chiba, Japan; 3 Cognition and Action Research Group, Human Technology Research Institute, National Institute of Advanced Industrial Science and Technology (AIST), Aist Tsukuba Central 6, Ibaraki, Japan; 4 Application Development Office, Hitachi Medical Corporation, Chiba, Japan; University of Montreal, Canada

## Abstract

A cross-modal association between somatosensory tactile sensation and parietal and occipital activities during Braille reading was initially discovered in tests with blind subjects, with sighted and blindfolded healthy subjects used as controls. However, the neural background of oral stereognosis remains unclear. In the present study, we investigated whether the parietal and occipital cortices are activated during shape discrimination by the mouth using functional near-infrared spectroscopy (fNIRS). Following presentation of the test piece shape, a sham discrimination trial without the test pieces induced posterior parietal lobe (BA7), extrastriate cortex (BA18, BA19), and striate cortex (BA17) activation as compared with the rest session, while shape discrimination of the test pieces markedly activated those areas as compared with the rest session. Furthermore, shape discrimination of the test pieces specifically activated the posterior parietal cortex (precuneus/BA7), extrastriate cortex (BA18, 19), and striate cortex (BA17), as compared with sham sessions without a test piece. We concluded that oral tactile sensation is recognized through tactile/visual cross-modal substrates in the parietal and occipital cortices during shape discrimination by the mouth.

## Introduction

A cross-modal association between somatosensory tactile sensation and parietal and occipital cortical activities during Braille reading in blind subjects was initially reported in 1996 by Sadato et al. [1], while multisensory processing of tactile and visual convergence were also later reported in sighted [2]–[7] and blindfolded [2], [8]–[10] healthy control subjects. Regarding cross-modal object recognition, bottom-up and top-down corticocortical mechanisms have been speculated [11]–[12]. Although an individual is not able to visualize the inside of their own oral cavity, it is possible to properly chew a food bolus. However, if that multimodal recognition in the mouth is disrupted, ordinary chewing may not be possible without sustaining an oral injury. In the field of dentistry, oral shape discrimination has been applied to examine oral stereognosis and chewing efficacy in aged edentulous patients [13]–[18], as well as the relationship between oral stereognosis and chewing activity [13]–[15], [19], and the effects of tooth loss and denture wearing [16], [20]–[23].

There are methodological differences among the examinations utilized to examine oral stereognosis with an oral shape discrimination task performance, mostly in terms of presenting [13]–[16] or not presenting [19], [21] the test pieces to the subject prior to the trial, and with [24] and without [13]–[16], [19], [21] subject rehearsal of the procedures preceding the sessions. As a result, no unified procedure for examination of oral stereognosis has been established, and there are no standard evaluations of the differences between presenting and not presenting the test pieces prior to the task.

The neural background involved in oral stereognosis also remains unclear. Several studies have investigated oral shape discrimination and its effects on cortical activities, including Fujii et al. [25], who initially reported prefrontal cortex activation, whereas there are no known reports of parietal and occipital cortical activities during oral shape discrimination. Yamamura et al. [26] reported cortical activation during shape discrimination by the mouth in the prefrontal and visual cortices. Nevertheless, currently available findings are contradictory and inconsistent, especially in regard to activation of the occipital cortex during shape discrimination by the mouth.

In the present study, we investigated whether the occipital cortex is activated during shape discrimination by the mouth using functional near-infrared spectroscopy (fNIRS). This technology is applicable in clinical situations and previous studies have detected visual cortex activation using fNIRS [27]–[29].

In this study, we presented the test pieces to the subjects and also allowed them to rehearse after receiving an explanation prior to all sessions of shape discrimination by the mouth. Our experiments consisted of 3 different sessions; resting prior to the trial (REST), discrimination without a test piece in the mouth (SHAM), and shape discrimination with individual test pieces in the mouth (SHAPE). The results of those sessions were used to determine baseline, control, and experimental values, respectively.

The purpose of this study was threefold. First, we attempted to identify the neural background related to reference imagery by explaining the test pieces and allowing rehearsal before the trials in the SHAM session. We also determined the effects of the association of target imagery with the test pieces in the mouth during the SHAPE session with reference imagery based on explanations of the test pieces and rehearsal preceding the trials during the SHAM session. Finally, the effects of target imagery with test pieces in the mouth on occipital cortex activation were compared between the SHAPE and SHAM sessions. From our findings, related neural substrates in the occipital cortex and their functional significance for oral shape discrimination are discussed.

## Materials and Methods

### Subjects

Eleven healthy, male, right-handed subjects aged 28.4±5.8 years (mean ± SD) were enrolled. All provided written informed consent for participation in the study, which was approved by the Ethics Committee of Nihon University School of Dentistry at Matsudo. All subjects were mentally healthy, as indicated by a score of ≤7 on the Hospital Anxiety and Depression Scale [30].

### Experimental procedures

**Figure 1:**
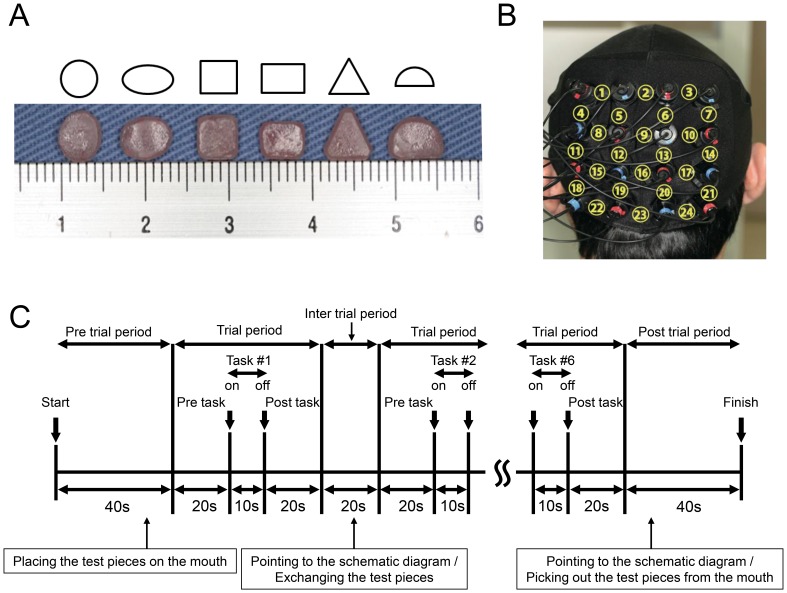
Experimental design and positions of near-infrared spectroscopy (NIRS) probes. A: Photograph and schematic diagram of the test pieces (circle, ellipse, square, rectangle, triangle, semicircle) used for shape discrimination by the mouth (oral shape discrimination; SHAPE). B: Position of NIRS probes and corresponding channel numbers. C: Timeline of a single session, which was composed of 6 trials. Prior to the session, subjects were shown the test pieces and a schematic diagram of the test pieces, received an explanation of the procedure, and were allowed to rehearse the task. During the SHAPE trials, a test piece was placed on the tongue of the subject. The subject was instructed to take this into their mouth and remain quiet until receiving a verbal cue (pre-trial period). At the verbal cue, the subject was instructed to explore the test piece in their mouth without biting (task period, 10 seconds). After 10 seconds, another verbal cue was given and the subject was instructed to remain quiet for 20 seconds (post-task period). Next, the subject was shown a schematic diagram of the 6 test pieces and asked to point to their answer after the test piece was removed from the mouth during the inter-trial and post-trial periods. Each trial was performed a total of 6 times, once for each shape. During the SHAM trials, the subjects performed the same protocol but without the test pieces.

The experiment was performed in a quiet room with the subject comfortably seated. A screen was positioned around the subject to block extra visual information entering their field of view. The session required the subject to discriminate the shape of test piece made of autopolymer resin (UNIFAST3, GC, JAPAN) that was placed in the mouth (oral shape discrimination; SHAPE). There were 6 test pieces with different shapes; circle, ellipse, square, rectangle, triangle, and semicircle [16] (Fig. 1A). Prior to the session, each subject was shown the 6 test pieces and a schematic diagram, received an explanation of the procedure, and was allowed to rehearse the task [16].

During the pretrial period, the investigator approached the subject from the rear so as not to enter their field of view, then instructed them to open their mouth and placed a test piece on the tongue using tweezers. The subject was instructed to be quiet until given a verbal cue (pretrial and pre-task periods), after which they were instructed to explore the test piece in their mouth without biting (task period, 10 seconds). After 10 seconds, they were given a verbal cue to stop the oral exploration and instructed to be quiet again (post-task period). After 20 seconds, the test piece was removed from the mouth, then the subject was shown a schematic diagram of all 6 test pieces and asked to point to the one they thought had been used. The trials was performed 6 times in 1 session, using a different shaped piece in each trial. In addition, the SHAM and REST sessions were conducted in a similar manner, but with no test piece placed in the mouth. During the SHAM session, the subject was instructed to move their tongue in the oral cavity, then point toward the schematic diagram after the experimenter had imitated removing the test piece. During the REST session, there was no task performance and the subject was instructed to remain quiet. The SHAPE and SHAM sessions were performed in random order.

### NIRS measurements

Occipital lobe activity was assessed during the pre-task, task, and post-task periods using a 24-channel NIRS device (ETG-100, Hitachi Medical Co., Chiba, Japan) that utilizes 2 wavelengths of near-infrared light (780 and 830 nm) [31]. The distance between the pairs of detector probes was 3.0 cm and the device was set to measure from points associated with the surface of the cerebral cortices [32]–[33]. The probes were fitted with 4×4 thermoplastic shells and placed at the center of the bottom line of shell, which was positioned on the inion (Iz) according to the international 10–20 system [34] (Fig. 1B). The distance between the tip of the probe and bottom of the shell was 1.0 cm. Functional NIRS was used to determine relative changes in the concentration of oxygenated-hemoglobin ([oxy-Hb]), deoxygenated-hemoglobin ([deoxy-Hb]), and total hemoglobin. Fundamental Hb data were displayed and used as an index of Hb change as a scaled variable. Change in [oxy-Hb] was used as an indicator of change in regional cerebral blood volume, because that is more sensitive than [deoxy-Hb] as a parameter for measuring blood flow changes associated with brain activation [35], and has a stronger correlation with blood-oxygenation-level-dependent signals measured by fMRI [36]. The sampling interval was 0.1 seconds. During the measurements, the subject was instructed to open their eyes and gaze at a point forward. Each trial was repeated 6 times and data obtained were averaged using the ‘integral mode’ of the ETG-100 software for the SHAPE, SHAM, and REST sessions. The moving average method (moving average window, 5 seconds) was used to exclude short-term motion artifacts. The baselines for measurements were corrected using linear fitting [37], which was performed by connecting the pre-task baseline (mean of the last 20 seconds of the pre-task period) to the post-task baseline (mean of the last 20 seconds of the post-task period).

### Anatomical identification of NIRS channels

The coordinates of all probe positions and anatomical landmark positions (Nz, Iz, A1, A2, Cz) were obtained using a 3-dimensional digitizer (3SPACE ISOTRAK2, Polhemus, US) and transcribed into Montreal Neurological Institute standard brain space [38]–[39] using probabilistic registration [40]–[41]. The probe positions were then projected onto the cortical surface. Next, the anatomical localization corresponding to the probe coordinates was identified using Platform for Optical Topography Analysis Tools (POTATo, Hitachi, Japan) with reference to Automated Anatomical Labeling [42].

### Statistical analysis

The value for [oxy-Hb] was calculated every 1 second and compared between the SHAM and REST, SHAPE and REST, and SHAPE and SHAM sessions using paired t-tests implemented with a plug-in-based analysis platform that runs on MATLAB (The MathWorks Inc.), and values showing p<0.05 were considered to be significantly different. A topographical representation of significant (p<0.05) channels every 1 second was projected onto the occipital cortical surface of a Montreal Neurological Institute standard brain space [43]–[44] using a 3-dimensional composite display unit (version 2.41, Hitachi Medical Co. Chiba Japan) [45].

It has been shown that as the number of items being examined increases, so does the risk of type 1 errors [46]. Thus, in order to avoid such errors, two-way repeated-measures ANOVA was applied and multiple comparisons using Tukey's method for the time course of averaged data of [oxy-Hb] every 1 second between the task periods of the SHAM and REST, SHAPE and REST, and SHAPE and SHAM sessions were performed. P-values less than 0.05 were considered to indicate significance.

## Results

### 1. Anatomical identification of NIRS channels

**Figure 2:**
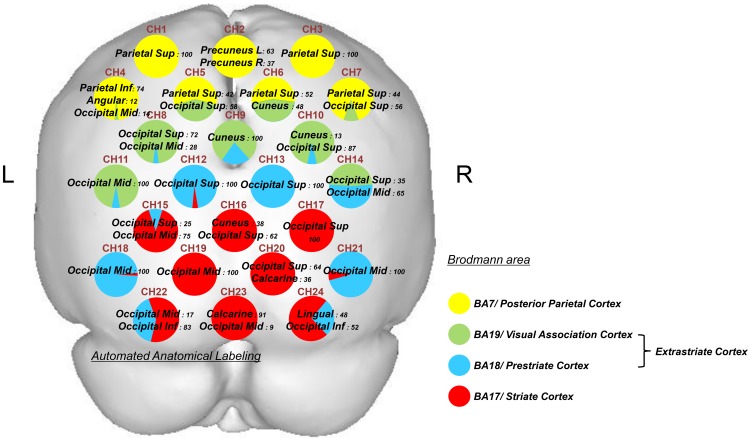
Anatomical identification of near-infrared spectroscopy channels. Coordinates for all probe and anatomical landmark positions (Nz, Iz, A1, A2, and Cz) were obtained using a 3-dimensional digitizer. Probabilistic registration was used to transcribe the measuring points of each subject according to the protocol of the Montreal Neurological Institute, and these points were projected onto the cortical surface. Anatomical localization was identified using the Platform for Optical Topography Analysis Tools, with reference to the Automated Anatomical Labeling system. Yellow indicates the posterior parietal cortex (BA7), green BA19 of the extrastriate cortex, blue BA18 of the extrastriate cortex, and red the striate cortex (BA17). Each circle corresponds to a channel and the pie chart within each circle shows the percentage of areas in that channel.

Anatomical identification of the NIRS channels is shown in Figure 2. Those channels were localized in the posterior parietal cortex (BA7), extrastriate cortex (BA18, 19), and striate cortex (BA17).

### 2. Grand averaged waveforms and topographical maps

**Figure 3:**
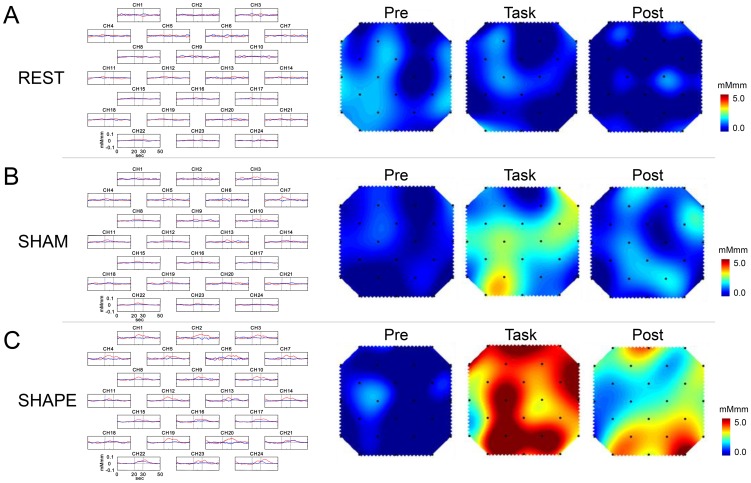
Grand average waveforms and topographical maps. Left: Grand average change in oxygenated-hemoglobin concentration ([oxy-Hb], red line) and deoxygenated-hemoglobin concentration ([deoxy-Hb], blue line) during (A) REST, (B) SHAM, and (C) SHAPE) sessions for each of the 24 measurement channels. The x-axis indicates time (s) and y-axis indicates hemodynamic change (mMmm). Grey vertical lines at 20 and 30 seconds indicate the start and end of the 10-second task period. Right: Topographical maps for changes in [oxy-Hb] in the 10-second period preceding the task period (Pre), the 10-second task period (Task), and the 10 seconds following the task period (Post) during the (A) REST, (B) SHAM, and (C) SHAPE sessions. There was no change in [oxy-Hb] throughout the REST session (A), while there was a slight increase during the task period in the SHAM session (B), and a marked increase during and following the task period in the SHAPE session (C).

The grand averaged waveforms for changes in [oxy-Hb] and [deoxy-Hb] during the REST, SHAM, and SHAPE sessions are shown in Figures 3A, B, and C, respectively. Figure 3 shows the topography of change in [oxy-Hb] during the pre-task, task, and post-task periods in the REST, SHAM, and SHAPE sessions. In the REST sessions, the change in [oxy-Hb] was scant throughout the session and there was no apparent change in [oxy-Hb] in the occipital lobe (Fig. 3A). In the SHAM session, there was a slight increase in [oxy-Hb] in the extrastriate cortex (BA18, 19) and striate cortex (BA17) during the task period (Fig. 3B), whereas there were marked increases in [oxy-Hb] in the posterior parietal cortex (BA7), extrastriate cortex (BA18, 19), and primary visual cortex (BA17) during the task and post-task periods in the SHAPE session (Fig. 3C).

### 3. Cross-sectional statistical topographies

#### 3-1. Comparison between SHAM and REST sessions

**Figure 4:**
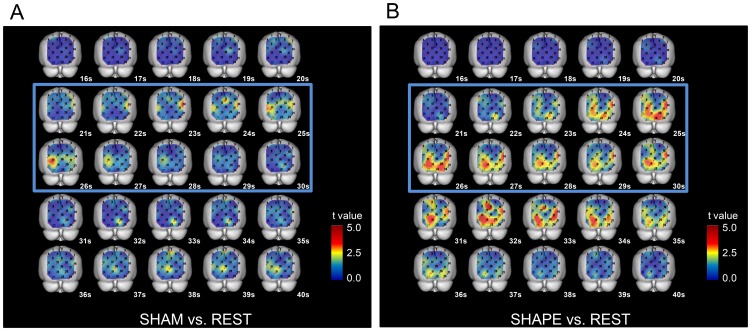
Cross-sectional statistical topographies showing differences in change in oxygenated hemoglobin concentration ([oxy-Hb]). A. Comparison between SHAM and REST sessions. The values for [oxy-Hb] during the task and post-task periods of the SHAM session were slightly but significantly increased (p<0.05, paired t-test) as compared to those of the REST session in the posterior parietal cortex (BA7), extrastriate cortex (BA18, 19), and primary visual cortex (BA17). B. Comparison between SHAPE and REST sessions. The values for [oxy-Hb] during the task and post-task periods of the SHAPE session were significantly increased (p<0.05, paired t-test) as compared to those of the REST session in the somatosensory association cortex (BA7), extrastriate cortex (BA18, 19), and striate cortex (BA17).

The values for [oxy-Hb] in the task and post-task periods of the SHAM session were slightly but significantly increased (p<0.05, paired t-test) as compared to those of the REST session in the posterior parietal cortex (BA7), extrastriate cortex (BA18, 19), and primary visual cortex (BA17) (Fig. 4A).

#### 3-2. Comparison between SHAPE and REST sessions

In the posterior parietal cortex (BA7), extrastriate cortex (BA18, 19), and striate cortex (BA17), the values for [oxy-Hb] in the task and post-task periods of the SHAPE session were significantly increased (p<0.05, paired t-test) as compared to the REST session (Fig. 4B).

### 4. Cross-sectional statistical topographies of [oxy-Hb] in the SHAPE and SHAM sessions

**Figure 5:**
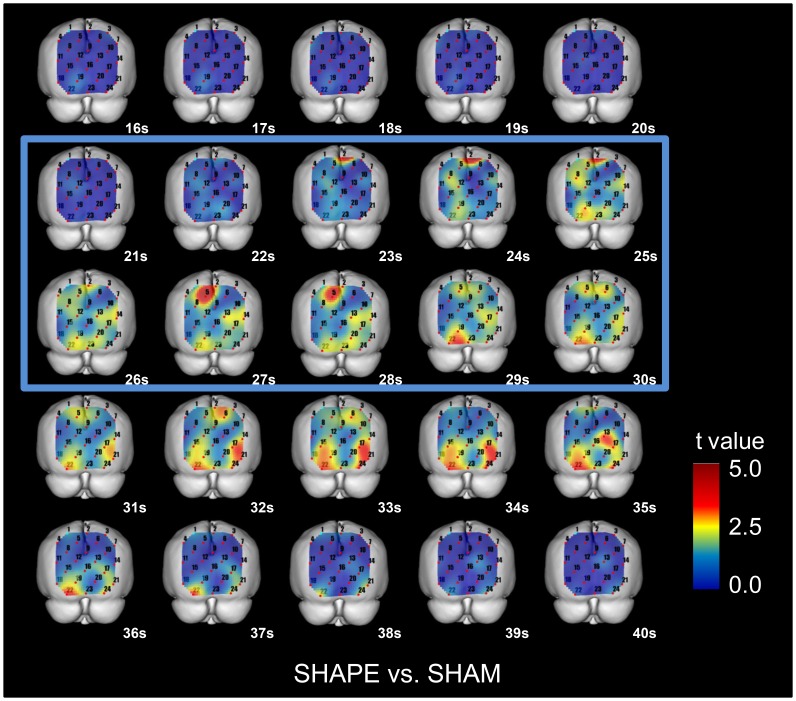
Cross-sectional statistical topographies showing differences in oxygenated-hemoglobin concentrations ([oxy-Hb]) between SHAPE and SHAM sessions. The values for [oxy-Hb] during the task period of the SHAPE session were significantly increased (p<0.05, paired t-test) as compared to the SHAM session in the posterior parietal cortex (BA7), extrastriate cortex (BA18, 19), and striate cortex (BA17).

In the posterior parietal cortex (BA7), extrastriate cortex (BA18, 19), and striate cortex (BA17), the values for [oxy-Hb] in the task and the post-task periods of the SHAPE session were significantly increased (p<0.05, paired t-test) as compared to the SHAM session (Fig. 5).

### 5. Temporal changes in [oxy-Hb] in REST, SHAM, and SHAPE sessions

**Figure 6:**
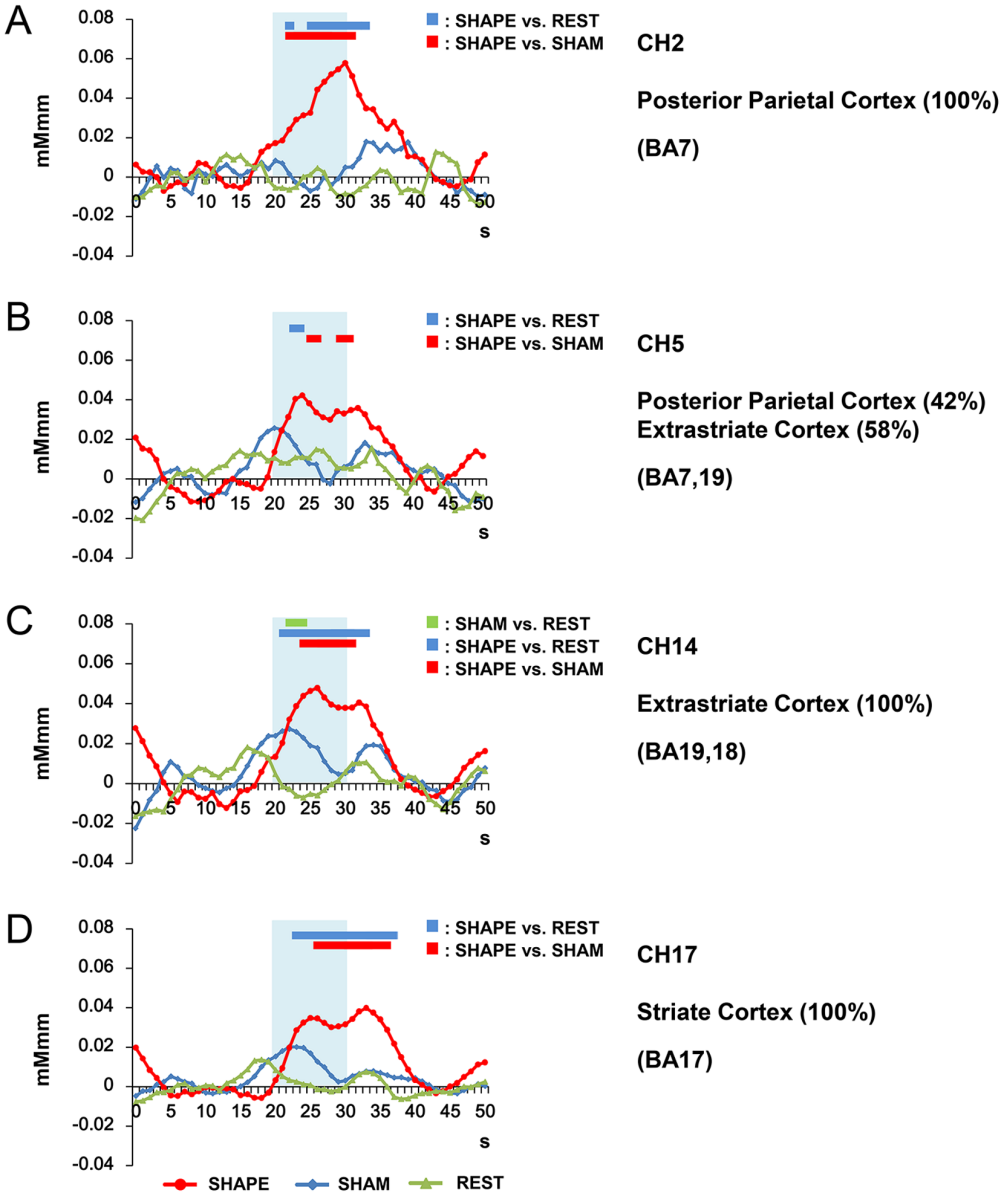
Temporal changes in oxygenated-hemoglobin concentration ([oxy-Hb]). Shown are changes in [oxy-Hb] at channels 2 (A; PPC/BA7), 5 (B; PPC/extrastriate cortex, BA19), 14 (C; extrastriate BA18), and 17 (D; striate cortex) during the REST, SHAM, and SHAPE sessions. Significant differences between SHAM and REST are indicated by a green bar, between SHAPE and REST by a blue bar, and between SHAPE and SHAM by a red bar. Statistical comparisons were conducted using two-way repeated-measure (ANOVA) of time and condition (SHAPE, SHAM, REST), followed by Tukey's post-hoc test. The level of significance was set at p<0.05. Significant differences between SHAPE and SHAM, and SHAPE and REST were clearly present in the posterior parietal, extrastriate, and striate cortices during the task and post-task periods. Blue shading indicates the task period.

Following the statistical comparison using paired t-tests between values obtained in the SHAPE and REST sessions (Fig. 5), we performed two-way repeated-measure ANOVA of data obtained from the channels, which showed a significant increase in [oxy-Hb] presented in Fig. 6. Our findings showed a significant correlation between time and task (p<0.05). In addition, post-hoc tests indicated statistically significant differences between the SHAPE and SHAM, SHAPE and REST, and SHAM and REST sessions (p<0.05, Tukey's method for multiple comparisons).

Significant differences regarding changes in [oxy-Hb] during the task periods were seen in channels CH2 (posterior parietal cortex/precuneus, BA7) (Fig. 6A), CH5 (posterior parietal cortex/exstrastriate cortex/BA7, 19) (Fig. 6B), CH14 (extrastriate cortex, BA18, 19) (Fig. 6C), and CH17 (striate cortex/BA17) (Fig. 6D) between the SHAPE and REST, and SHAPE and SHAM sessions (p<0.05, Tukey's method for multiple comparisons). Furthermore, there was a significant difference noted in channel CH17 (BA17), which presented a more long-lasting activation of [oxy-Hb], between the SHAPE and SHAM, and SHAM and REST sessions (Fig. 6D) (p<0.05, Tukey's method for multiple comparisons).

## Discussion

In the present study, we obtained the following notable results. Activation was seen in the posterior parietal cortex (BA7), extrastriate cortex (BA18, BA19), and striate cortex (BA17) during the SHAM control session without test pieces as compared to the REST session. We also noted activation in the PPC (BA7), extrastriate cortex (BA18, 19) and striate cortex (BA17) in the SHAPE session with test pieces as compared to the REST session. Furthermore, specific activation was seen in the PPC (BA7), extrastriate cortex (BA18, 19) and striate cortex (BA17) in the SHAPE session as compared to the SHAM session.

Based on our results, we will first discuss neural substrates related to reference imagery such as shape memory and imagery, which were possibly activated by our explanation of the test pieces and rehearsal preceding the sessions in the present subjects. In addition, we discuss verification of target imagery arising from manipulation of the test piece in the mouth. Finally, we speculate that the effects of target imagery facilitate parietal and occipital cortex activities.

### Effects of SHAM session as compared to REST

We found activation in the posterior parietal cortex (BA7), extrastriate cortex (BA 18, 19), and striate cortex (BA17) during the SHAM discrimination sessions without test pieces as compared to the REST session. As noted above, preceding performance of the REST, SHAM, and SHAPE sessions, the participants were presented the test pieces and rehearsed shape discrimination without a test piece in the mouth. Therefore, even with no test pieces to manipulate, shape memory and imagery may have activated the parietal and occipital cortices during the SHAM session.

Mayer et al. [47] reported a common neural substrate for visual working memory and attention in the prefrontal cortex, visual, and parietal areas. Also, Kosslyn et al. [48] and Stokes et al. [49] noted the involvement of primary visual cortex activation in visual imagery, while Cavanna et al. [50] reported visual memory induced in the PPC, and Todd et al. [51], Sathian et al. [52], and Parra et al. [53] found that was activated in the PPC and extrastriate cortex during working memory activation. Also, Ven et al. [54] applied TMS to examine visual cortex disruption resulting in memory interference. Furthermore, Jonides et al. [55] reported that rehearsal of information entering working memory from the visual external world is processed by structures in the parietal and temporal lobes specialized for perceptual processing, and these same structures remain active when the stimulus is removed for a brief duration. From these and our findings, it is speculated that activations of the posterior parietal cortex (BA7), extrastriate cortex (BA 18, 19), and striate cortex (BA17) during the SHAM control session could be elicited by shape imagery and/or shape memory introduced by the instructions and rehearsals preceding the sessions.

### Effects of SHAPE session as compared to REST

Activation in the posterior parietal cortex (BA7), extrastriate cortex (BA18, 19), and striate cortex (BA17) in the SHAPE session as compared with the REST session was observed in our study.

Ptito et al. [2] reported that tactile stimulation of the tongue evoked posterior parietal cortex and extrastriate cortex activities, while James et al. [3] and Sathian et al. [56] found exstrastriate cortex activation during shape discrimination by fingers trials. Nakashita et al. [57] also reported tactile-visual integration in the posterior parietal cortex and Pasalar et al. [58] noted that application of TMS to the posterior parietal cortex disrupted shape discrimination. Furthermore, Hannula et al. [59] and Zangaladze et al. [60] applied TMS stimulation to the primary somatosensory cortex (S1) and confirmed disruption in tactile temporal discrimination.

When considering tactile shape discrimination from the neural substrate in bottom-up, top-down, and cross-modal tactile/visual association, it is also speculated that bottom-up oral tactile sensation may undergo a top-down processing from posterior parietal cortex (BA7) toward the extrastriate and striate cortices [11]–[12].

Furthermore, in consideration of the aspect of behavior during the SHAPE session, we observed that the subjects likely rotated the test piece with the tongue and lips in order to understand the angular gradient. This manipulation was sufficient for mental rotation by the test piece in the mouth, which induced activation of the posterior parietal cortex and extrastriate cortex. Keeher et al. [61] reported that the superior parietal cortex exhibited a positive linear relationship hemodynamic response and degree of rotation, while Cohen et al. [62] also found activation of the posterior parietal cortex during mental rotation of shape. Taken together, target imagery including bottom-up oral tactile sensation during shape discrimination by the mouth may be associated with the posterior parietal cortex (BA7), extrastriate cortex (BA 18, 19), and striate cortex (BA17), and these neural substrates in parietal and occipital lobes also may play a role in mental rotation by the test piece in the mouth.

### Parietal and occipital cortex activation related to presence or absence of test piece in mouth during shape discrimination

In the present trials, we noted significant activation in the posterior parietal cortex (BA7), extrastriate cortex (BA18, 19), and visual cortex (BA17), based on the statistically significant differences between the SHAPE and SHAM sessions. Furthermore, the activation was identical with that seen in the SHAM session. These results suggest that target imagery induced by the test piece in the mouth may have activated the identical cortical regions in the posterior parietal cortex (BA7), extrastriate cortex (BA18, 19) and striate cortex (BA17) as seen in the SHAM session. Thus, we consider that target imagery including bottom-up oral sensations facilitates shape imagery and shape memory related to cortical activation in those 3 areas. Our findings also revealed long-lasting activation of [oxy-Hb] during the post-task periods in the SHAPE and SHAM, and SHAM and REST sessions (Fig. 6D). Visual cortex activation in the post-task period is thought to occur as storage of shape memory during recognition by shape discrimination.

Ptito et al. [2] reported activation in the inferior parietal, precuneus, and prefrontal cortices, but not cuneus by tongue tactile stimulation in healthy control subjects using a tongue display unit (TDU). Considering the differences in data between that study obtained by passive stimulation to the tongue and the present study obtained by shape discrimination in the mouth, the latter method is thought to be more effective for verification of the test pieces, as well as shape and memory imagery [48], [54]–[55] and mental rotation [61]–[62]. This is the first known study to report parietal and occipital cortex activation during shape discrimination by the mouth.

### Clinical implications

Oral examinations using shape discrimination have been conducted in dental research, especially in the field of prosthodontics, in order to elucidate chewing ability and oral sensitivity, and the relationships between chewing ability and oral sensitivity. However, as noted above, the procedures used for oral shape discrimination in past investigations are inconsistent in regard to presentation of the test pieces preceding the shape discrimination task. A standard method for oral discrimination is necessary to provide reliable findings from investigations of both healthy controls and patients.

In the present study, parietal and occipital cortical activations during shape discrimination by the mouth were observed. In contrast, Fujii et al. [25] reported activation in the prefrontal cortex but not the occipital cortex. According to Lacey and Sathian [11], haptic perception of a familiar shape uses visual object imagery via top-down paths from the prefrontal and parietal areas, whereas haptic perception of an unfamiliar shape may use special imagery processes and involve bottom-up pathways from the somatosensory cortex. Therefore, we speculate that reference imagery with explanation of the test pieces may produce familiarity with the pieces, which may facilitate top-down processing from the prefrontal and parietal cortices to parietal and occipital cortical activities. Because of familiarity with the test pieces, our data may be inconsistent with prior research results presented by Fujii et al. [25].

Additional investigations are necessary for better understanding of the effects of reference imagery in order to evaluate oral sensory perception ability in healthy subjects, as well as aged patients who are edentulous, and patients with movement disorders or psychiatric issues. Our results warrant further study regarding how reference imagery modulates the activation of neural substrates induced by target imagery during shape discrimination by the mouth, and how deprivation of intraoral tactile sensation modulates the neural substrates in the parietal and occipital lobes.

### Summary and conclusions

The following results were obtained in this study. After recognition of the shape of the test piece, shape discrimination without the piece in the mouth activated the extrastriate cortex (BA18, 19) and striate cortex (BA17). Furthermore, shape discrimination with the test piece activated the posterior parietal cortex (BA7), extrastriate cortex (BA18, 19), and striate cortex (BA17), and specifically activated the posterior parietal cortex (BA7), extrastriate cortex (BA18, 19) and striate cortex (BA17), as compared to the SHAM session without the test pieces. We concluded that parietal and occipital cortical areas may play important roles in intraoral somatosensory shape discrimination.
